# Virtual drug repurposing study against SARS-CoV-2 TMPRSS2 target

**DOI:** 10.3906/biy-2005-112

**Published:** 2020-06-21

**Authors:** Serdar DURDAĞI

**Affiliations:** 1 Department of Biophysics, Computational Biology and Molecular Simulations Laboratory, School of Medicine, Bahçeşehir University, İstanbul Turkey

**Keywords:** Drug repurposing, TMPRSS2, COVID19, SARS-CoV-2

## Abstract

Currently, the world suffers from a new coronavirus SARS-CoV-2 that causes COVID-19. Therefore, there is a need for the urgent development of novel drugs and vaccines for COVID-19. Since it can take years to develop new drugs against this disease, here we used a hybrid combined molecular modeling approach in virtual drug screening repurposing study to identify new compounds against this disease. One of the important SARS-CoV-2 targets namely type 2 transmembrane serine protease (TMPRSS2) was screened with NPC’s NIH small molecule library which includes approved drugs by FDA and compounds in clinical investigation. We used 6654 small molecules in molecular docking and top-50 docking scored compounds were initially used in short (10-ns) molecular dynamics (MD) simulations. Based on average MM/GBSA binding free energy results, long (100-ns) MD simulations were employed for the identified hits. Both binding energy results as well as crucial residues in ligand binding were also compared with a positive control TMPRSS2 inhibitor, Camostat mesylate. Based on these numerical calculations we proposed a compound (benzquercin) as strong TMPRSS2 inhibitor. If these results can be validated by in vitro and in vivo studies, benzquercin can be considered to be used as inhibitor of TMPRSS2 at the clinical studies.

## 1. Introduction

Coronaviruses (CoVs) belong to a single-stranded RNA (positive-sense) virus family which is encapsulated by a membrane envelope (Durdagi et al., 2020). There are 4 common types of coronavirus (alpha, beta, gamma, and delta) and cause mild upper respiratory tract diseases (McKee et al., 2020). Human beta-coronaviruses are highly pathogenic (Zhang et al., 2020). The SARS (Severe Acute Respiratory Syndrome)-CoV was appeared in Guangdong in China and infected 8096 people worldwide in 2002–2003. The fatality rate was around 10% (i.e. 774 deaths). In 2012, the MERS (Middle East Respiratory Syndrome)-CoV infected about 2500 people and the fatality rate was 36% (Blanco-Melo et al., 2020). Currently, the world suffers from a novel coronavirus SARS-CoV-2 that causes 2019 coronavirus disease (COVID-19). Like SARS and MERS-CoVs, SARS-CoV-2 mainly affects the lower respiratory tract (Blanco-Melo et al., 2020). It is characterized by a number of symptoms such as fever, cough, diarrhoea, and general weakness (Blanco-Melo et al., 2020). In more serious cases, it causes acute respiratory distress syndrome and lung damage leading to inflammation and pneumonia. On the 11th of March 2020, the WHO declared the COVID-19 as a pandemic (Hoffmann et al., 2020). It affected the lives of hundreds of millions of people as a result of compulsory isolation and quarantines in the world (Sanders et al., 2020). SARS-CoV-2 targets host cells via Spike protein that binds to the angiotensin-converting enzyme 2 (ACE2) receptor. The virus then uses host cell receptors (i.e. type 2 transmembrane serine protease (TMPRSS2)) and endosomes to enter the cells. TMPRSS2 facilitates cell entry of SARS-CoV-2 through the Spike protein. After entering the host cell, viral proteins are synthesized that encode for the replicase-transcriptase complex (Mousavizadeh and Ghasemi 2020). Viral RNA was then synthesized by RNA-dependent RNA polymerase (Durdagi et al., 2020). The genome of SARS-CoV-2 encodes for different structural and nonstructural proteins (Sanders et al., 2020). The important ones for drug development studies are Main Protease, and RNA-dependent RNA polymerase (Cao et al., 2020). Currently these proteins are mainly targeted for drug screening and drug repurposing studies. 

It is noteworthy that the method known as drug repurposing has become less costly in terms of both time and resources. In our laboratory, the use of molecules in different indications has been investigated with the studies we have done in this field in recent years (Durdagi et al., 2018; Is et al.,2018; Tutumlu et al., 2020). Since the most of the preclinic and clinical studies including pharmacokinetic and toxicological studies of approved or compounds in clinical investigation phases have been already tested, they require less time to make them suitable for new indications. The toxicity and ADME (absorption, distribution, metabolism and excretion) studies which need long time to complete are not required as the molecules considered in drug repositioning pass these stages already that should be applied before and have well-defined profiles. Therefore, their use in epidemics is more suitable than new molecules that have never been tested. Hence, in the current study we performed a virtual drug repurposing study. The small molecules from NCGC-NIH Chemical Genomics Center Pharmaceutical Collection (i.e. NPC library) were used in virtual screening studies at the active site of developed TMPRSS2 model target protein.

## 2. Materials and methods

We downloaded and prepared 7922 small compounds from NPC library (https://tripod.nih.gov/npc/). Before the virtual screening, to remove nonspecific compounds, we performed some filtration criteria. Such as molecular weights of compounds which are more than 1000 g/mol and smaller than 100 g/mol were removed from the library. The compounds that have more than 100 rotatable bonds or hydrogen bond acceptor and donor number that is higher than 10 were also removed. Thus, the total number of molecules were decreased to 6654 before the docking simulations. These compounds were prepared with LigPrep module (LigPrep, Schrodinger v.2017) of Maestro molecular modeling package. Since the crystal structure of the TMPRSS2 was not available, we performed homology modeling study. Swiss Model was used in homology modeling study (swissmodel.expasy.org). The 5CE1 PBB coded serine protease hepsin was used as template structure. Sequence identity between template and target proteins was 35.2%. Structural assessment studies on the developed model have been performed. There was no residue at the unfavourable region at the Ramachandran’s plot and protein reports showed no steric clashes, bond length or bond angle deviations at the structure, thus it was suitable to use as target protein (Supplementary Materials, Figure S1). Before the docking, modelled target protein was prepared using Protein Preparation module of Maestro molecular modeling package. In the determination of protonation states of residues at the target protein, PROPKA was used. A restrained optimization protocol was employed with OPLS3e force field for the target protein model using 0.3 Å convergence criteria. Docking was implemented with Standard Precision (SP) protocol of Glide using default settings. For the top-50 docking poses all-atom molecular dynamics (MD) simulations were performed to mimic physiological conditions using Desmond. Orthorhombic box was used with explicit water models (i.e. TIP3P) that have 10 Å thickness from the edges of the protein. 0.15 M NaCl was added to the simulation box to neutralize the system. The particle mesh Ewald method was used for long range electrostatic interactions. 9.0 Å cut-off was used for both electrostatic and van der Waals interactions. The temperature (310 K) and pressure (1.01325 bar) throughout the simulations were kept constant by Nose-Hoover thermostat (Evans and Holian, 1985) and Martyna-Tobias-Klein barostat (Martyna et al., 1994). The OPLS3e force field was used in MD simulations. 1000 trajectory frames for each system during the simulations were collected. Molecular Mechanics Generalized Born Surface Area (MM/GBSA) method was then employed for these trajectories and average MM/GBSA score for each studied compound was computed. The VSGB 2.0 solvation model in Prime module of Maestro was employed in MM/GBSA calculations. 

## 3. Results and discussion

**Figure 1:**
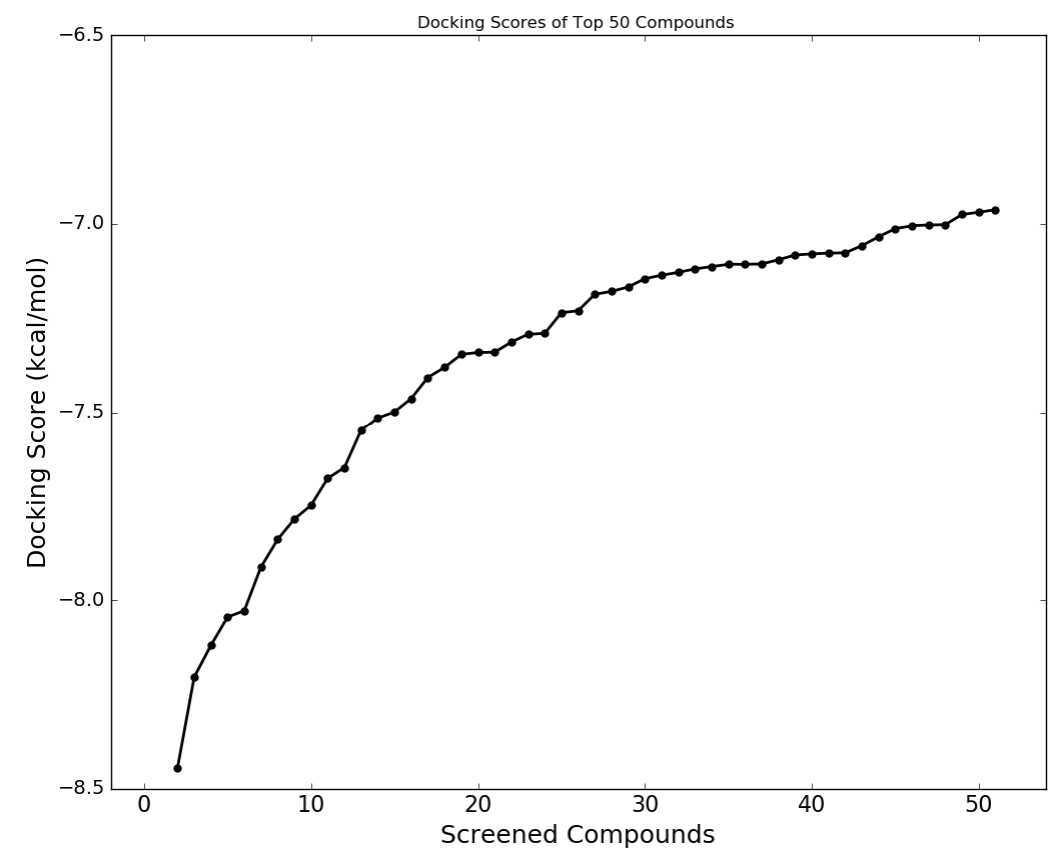
Docking scores of top-50 screened compounds from NPC database using Glide/SP.

**Figure 2:**
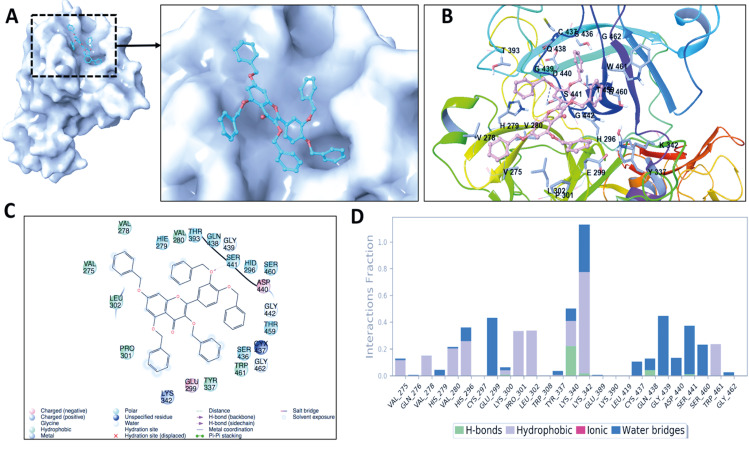
(A) Surface representation of benzquercin at the binding pocket of TMPRSS2. The last frame from 100-ns MD simulations was used. (B) 3D ligand interactions diagram of benzquercin at the TMPRSS2 site. (C) Corresponding interactions were also depicted with 2D. (D) Interaction fractions of binding pocket residues of TMPRSS2 with benzquercin throughout the MD simulations. Results show statistical results of collected 1000-trajectory frames throughout 100-ns MD simulations.

Nowadays thanks to the effort of NIH Chemical Genomics Center, it is possible to obtain electronic resources of collections of small molecules that have been registered. Combining this with the power of recent advances of ligand- and target-driven based virtual screening methods, it has been made easier to consider drug repurposing for any disease especially for complex diseases. We used homology model target TMPRSS2 structure and screened the prepared 6654 FDA approved drugs and compounds in clinical investigation phases from NPC database using Glide/SP. Docking scores were between –8.445 and 3.438 kcal/mol (Figure S2). Due to the limited flexibility of both the target protein and the screened ligands in docking, the identification of the compounds based on only docking scores in docking simulations can lead to false-positive results (Tutumlu et al., 2020). Moreover, although molecular docking simulations may provide an insight into protein/screened ligand interactions, it is always important to understand how these interactions are sustained throughout the performing MD simulations. Thus, we used top-50 docking scored compounds and employed initially short (10-ns) all-atom MD simulations in physiological and body temperature conditions. Docking scores of top 50 compounds were between –8.445 and –6.962 kcal/mol (see Figure 1 and Table at the Supplementary Materials). An in-house script was used to prepare simulation boxes and analyse MD simulations. Desmond was used in all MD simulations. Table represents the average MM/GBSA scores using the recorded 1000 frames (strided by 10 during the simulations in each system) of the top-50 ligands. In order to compare the docking scores and average MM/GBSA scores of identified hit compounds through our virtual screening protocol, we also used same screening protocol for a well-known TMPRSS2 inhibitor, Camostat mesylate. It is an approved small molecule compound for the treatment of pancreatitis in Japan. It is found that Camostat mesylate prevents SARS-CoV-2 cell entry by inhibition of host serine protease TMPRSS2. Its docking score was –5.444 kcal/mol at the binding pocket of the TMPRSS2. Its corresponding average MM/GBSA score was calculated as –65.514 kcal/mol. Figure S3 at the Supplementary Materials shows 2D ligand interactions diagram of Camostat mesylate at the binding site of SARS-CoV-2 TMPRSS2 target. Crucial residues were found as Val280, Asp435, Ser436, Gln438, Gly439, Ser441, Ser460, and Val473. Based on average MM/GBSA scores of short MD simulations, we selected 3 hits that have better scores than –70.0 kcal/mol, which are Benzquercin, Difebarbamate, and N-benzoyl-l-tyrosyl-paba. Long (100-ns) MD simulations were performed for these compounds and MM/GBSA scores were recalculated. Results showed that Benzquercin, N-benzoyl-l-tyrosyl-paba, and Difebarbamate have average MM/GBSA scores of –80.583, –56.162, and –66.567 kcal/mol, respectively. Results showed that especially Benzquercin (a flavonoid) has maintained the interactions with the binding pocket residues. Although average MM/GBSA score of Difebarbamate is slightly decreased at the long MD simulations compared to short simulation, it has still similar range of MM/GBSA score with positive control compound Camostat. Figure 2 represents the 2D and 3D ligand interactions diagram of Benzquercin at the binding site of TMPRSS2. The last frame from 100-ns MD simulations was used. Crucial residues at the target were found as His296, Glu299, Pro301, Leu302, Lys340, Lys342, Gly439, and Ser441 which share similar binding pocket residues with positive control Camostat. Based on interaction fractions analysis Lys342 has the highest interaction fraction value with the hit compound throughout the 100-ns simulations. The type of main interactions between the ligand and binding pocket residues were hydrogen bonds through water bridges and hydrophobic interactions. 

**Table:** Docking scores of top-50 compounds at the TMPRSS2 binding site. These compounds were initially used in short (10-ns) MD simulations. Table also shows average MM/GBSA scores of these compounds from derived 1000-trajectories throughout the simulations.

Compounds	Docking score (kcal/mol)	Average MM/GBSA(kcal/mol)
Reproterol	–8.445	–47.283
Ociltide	–8.204	–57.549
Theodrenaline	–8.120	–26.242
Hexafluronium bromide	–8.045	–67.775
Cliropamine	–8.028	–38.601
Dab-452	–7.912	–60.818
Fenoterol	–7.839	–45.418
Frakefamide	–7.785	–42.928
mitoxantrone	–7.749	–44.749
Adimolol	–7.677	–58.356
Mesuprina	–7.649	–45.323
Esorubicin	–7.549	–42.773
Arbutamine	–7.515	–56.584
Penimepicycline	–7.499	–44.513
(Alpha.s)-alpha-[alpha-(2,4-dimethyl-9h-pyrido(2,3-b)indol-9-yl)-p-tolyl]-n-((alpha.r)-alpha(hydroxymethyl)benzyl)cyclopentaneacetamide	–7.463	–64.419
1,2,3-Dihydro-5-benzo(b)thienyl-2-(4-phenylbutylamino)-1-propanol	–7.407	–44.191
Quinidine	–7.380	–48.078
Tilisolol	–7.346	–24.419
Primozida	–7.341	–66.157
Ad 810	–7.340	–37.037
Carbuterol	–7.313	–18.889
Pronetalol	–7.293	–24.264
Vanyldisulfamide	–7.290	–61.900
Difebarbamate	–7.235	–76.184
Compounds	Docking score (kcal/mol)	Average MM/GBSA(kcal/mol)
Piroxicillin	–7.230	–57.173
Dapoxetine	–7.187	–49.660
Etanterol	–7.179	–12.707
Zinterol	–7.167	–42.080
Amosulalol	–7.145	–45.639
Fenprostalene	–7.136	–57.394
Carazolol	–7.128	–37.720
Benzquercin	–7.119	–96.802
Naftopidil	–7.113	–56.904
Denopamine	–7.107	–56.310
Brefonalol	–7.107	–44.698
Nardeterol	–7.106	–49.928
3-(Cis-2,6-dimethylpiperidino)-n-(4 methoxybenzoyl)sidnonimine	–7.095	–52.087
Nordefrin	–7.083	–28.408
Ametantrone	–7.079	–49.325
Epicainide	–7.078	–60.527
Carteolol	–7.077	–29.853
Cinacalcet	–7.058	–52.311
N-benzoyl-l-tyrosyl-paba	–7.034	–71.183
Panamesine	–7.013	–56.305
Neraminol	–7.005	–45.562
Bucindolol	–7.003	–60.390
Unii-f6s9cmy4se	–7.002	–63.674
Giripladib	–6.975	–64.455
Pancopride	–6.969	–52.768
Nolinium bromide	–6.962	–51.067

Interestingly, benzquercin was also found as potent hit compounds in our previous virtual screening study using same protocol against another important cell-entry target of SARS-CoV-2 Spike/ACE2 (Durdagi et al., 2020). Its average score at the Spike/ACE2 interface was found as –70.810 kcal/mol. Hence, these studies showed that benzquercin may also act as dual inhibitor. It has been recognized that the treatment of “one target/one molecule” approach of complex diseases is not so effective. The usage of combined drugs is not also appreciated due to toxicity and/or undesirable drug-drug interactions. The recent and promising approach to these complex diseases including COVID-19 is instead to develop/identify unique compounds that act on multi-targets simultaneously which these targets are crucial in the studied disease. Thus, based on in silico results, benzquercin has this potential.

## 4. Conclusions

Molecular modeling studies, such as virtual screening, reduce the time required to set new targets for known drugs and also provide the advantage of being cost-effective (Shoichet et al., 2002; Durdagi et al., 2018 and 2020). In this study, a virtual drug repurposing study was performed to identify new compounds against TMPRSS2 which is an important target for the entry of the SARS-CoV-2 to the host cell. Thus, NPC small molecule library was screened initially by docking simulations. Based on docking scores, top-50 compounds were used in short MD simulations and their average binding free energies were calculated by MM/GBSA method. Selected hits were used in longer MD simulations and results showed that especially benzquercin maintains its interactions with the crucial residues throughout the simulations. If these results can be validated by experimental studies, benzquercin can be considered to be used as inhibitor of TMPRSS2 at the clinical studies.

## Acknowledgment

This study was supported by Bahçeşehir University, Scientific Research Projects Unit, Project No: BAP.2020-01.01

## Supporting Information

Figure S1Structural assessment of developed homology model structure of TMPRSS2. (A) Ramachandran’s plot. While filled spheres show each chiral residue at the target protein, filled triangles show Gly residues. (B) Local quality estimate of the derived model. (C) Normalized QMEAN4 score and its comparison with similar size of a set of PDB structures.
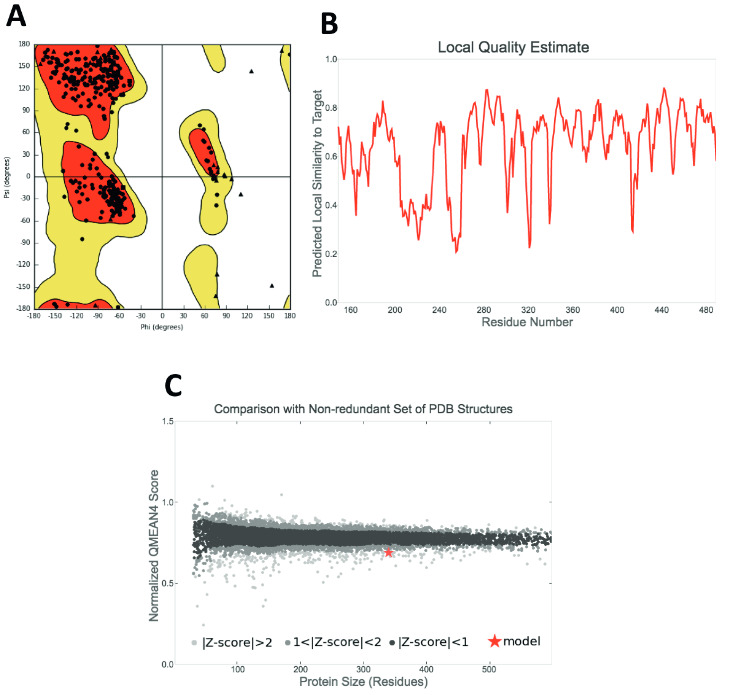

Figure S2Docking scores of all screened compounds at the binding pocket of TMPRSS2.
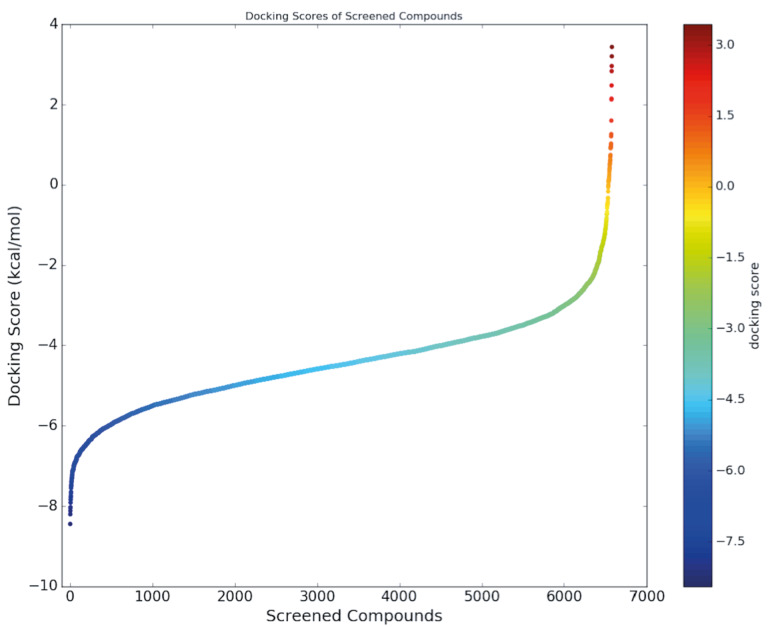

Figure S32D ligand interactions diagram of Camostat at the binding pocket of TMPRSS2.
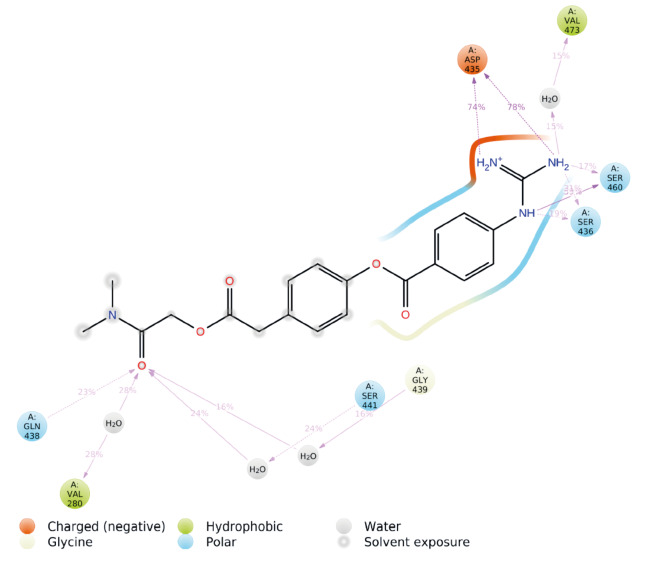

## References

[ref1] (2020). Imbalanced Host Response to SARS-CoV-. Drives Development of COVID-19, Cell.

[ref2] (2020). Comparative genetic analysis of the novel coronavirus (2019-nCoV/SARS-CoV-2) receptor ACE2 in different populations. Cell Discovery.

[ref3] (2018). Integration of Multi-scale Molecular Modeling Approaches with Experiments for the in silico Guided Design and Discovery of Novel hERG-Neutral Antihypertensive Oxazalone and Imidazolone Derivatives and Analysis of Their Potential Restrictive Effects on Cell Proliferation. European Journal of Medicinal Chemistry.

[ref4] (2020). Screening of Clinically Approved and Investigation Drugs as Potential Inhibitors of SARS-CoV-2 Main Protease and Spike Receptor-Binding Domain Bound with ACE2 COVID19 Target Proteins: A Virtual Drug Repurposing Study. ChemRxiv. doi: 10.

[ref5] (1985). The Nose–Hoover thermostat. Journal of Chemical Physics.

[ref6] (2020). SARS-CoV-2 Cell Entry Depends on ACE2 and TMPRSS2 and Is Blocked by a Clinically Proven Protease Inhibitor. Cell.

[ref7] (2018). Multidimensional Molecular Modeling Approaches and Application of Binary QSAR Models for Prediction of their Therapeutic Activity and Toxic Effects. ACS Chemical Neuroscience.

[ref8] (1994). Constant pressure molecular dynamics algorithms. The Journal of Chemical Physics.

[ref9] (2020). Candidate drugs against SARS-CoV-2. 2020 Apr 29]. Pharmacological Research.

[ref10] (2020). Genotype and phenotype of COVID-19: Their roles in pathogenesis. Journal of Microbiology, Immunology and Infection ISSN 1684-1182.

[ref11] (2020). COVID-19: Melatonin as a potential adjuvant treatment.. Life Sciences.

